# Remarkable Case of Palsy

**Published:** 1816-09

**Authors:** John Cross


*For the London Medical and Physical Journal.*

### *Remarkable Case of Palsy;* by John Cross, M. D.

GLASGOW, *April* 18, 1816.—Daniel M'Kechnie, petty officer in the royal navy, unmarried, aged thirty-five years, five feet five inches high, short neck, large head, sanguine temperament, and full habit; had been fifteen years in the service; nine of them in the West Indies, two in the Mediterranean, and the rest in home stations; had been always, while living in a cold climate, afflicted with piles, which uniformly disappeared on entering a warm climate; has been as far back as he recollects habitually costive. Since leaving the service, in March 1815, he has continued to eat and drink freely, and has not as yet engaged in any employment. He had been drinking pretty freely for about a week past with some of his shipmates who had paid him a visit, and, especially on the Monday, Tuesday, and Wednesday, had been deeply intoxicated. He had, however, refrained from the glass all the Thursday. In the evening, he stepped into a hardware shop to sit and converse with a few friends; and a little after eight o'clock, when the shop was about to be shut, was reaching out his hand for his hat, when in a moment he felt himself "quite overcome at the heart," and became for a minute completely blind. After swallowing a little cold water, he came to himself, soon felt refreshed, and, as he thought, quite recovered. However, on attempting to rise, his right foot gave way under him, the right arm hung powerless from the shoulder, and the face was drawn towards the left side. In this condition he was carried home. About three quarters of an hour after the attack I saw him. The right extremities, with the exception of very slight motion of the humerus and femur, were completely paralysed. His speech was almost unintelligible. His tongue, when thrust out of the mouth for my inspection, formed an arch, and universal tremor pervaded his frame. From the puffiness of the left temple, the pulsation of an artery could not be felt; the lancet was, therefore, put in at a venture. No blood having come, the external jugular vein was opened, and about ℥iii. of blood obtained with difficulty. A vein of the left arm was then opened, and ℥xviii. of blood allowed to flow in a full stream. While the blood was yet flowing, the tremors disappeared, and his articulation became more distinct. By and bye he became faintish, and was laid in bed. After recovering a little, he swallowed gr. xxv. of calomel. In two hours afterwards ℥xiv. more of blood were taken from the arm. This time he stood the bleeding better.

April 19. Morning.—His face is less drawn to a side, and and he can now move the whole right arm and leg, though with considerable difficulty. This improvement he dates from a most copious stool in the night-time. He has had two more stools since. All the three had a very dark colour, and most offensive smell. Two strong purgative pills were ordered. A blister was applied to the back of his neck. His diet was restricted to soups, and his drink to water.

Evening.—He has again lost the power of moving the right limbs, and the face is considerably more drawn to a side; ℥xviii. of blood were drawn in a large stream. By the time the arm was dressed he had recovered the same power which he possessed in the morning of moving the paralytic limbs, and felt "more lightsome about the heart."

April 20. Morning.—He continues to keep the ground he had gained last night. He had free purging early in the morning. He is ordered to swallow ℥i. of castor oil.

Evening.—He has considerably lost the power of moving the right arm, owing, as he thinks, to his having exposed it too much above the bed-clothes. His face is redder than usual; ℥xiv. of blood were drawn from the arm in a very large stream. Soon after the bleeding he acquired the power which he possessed in the morning of moving the arm; gr. xx. of calomel were given.

April 21.—Continues to improve; ℥vi. of Epsom salts, and ℥iii. of Glauber, are ordered to be melted in a chopin of cold water, and a wine-glassful to be given thrice a day. He is ordered to keep his bed, and to encourage gentle perspiration.

He continued to improve, and on the 27th was taken out of bed, for the purpose of ascertaining how he could walk, when to his agreeable surprise he walked across the room without making the slightest halt. He was ordered to take a wine-glassful of the solution every night and morning for at least two months.

At this date he is quite well, and going about as usual, but is persevering in the solution.

Instances there have been of spontaneous recovery from hemiplegia. But here the severity and the repetition of the paralytic attacks show the fatal tendency of the disease; while the marked benefit that repeatedly accompanied depletion, or followed at its back, establishes a manifest connection between the treatment and the recovery. A single case like the present, although it does not warrant any conclusion, nor of course deserve any comment, is in the mean time worthy of being recorded as a fact and as a hint.—*Annals of Philosophy*.

*Glasgow; May* 18, 1816.

